# A new species of *Siphoderina* Manter, 1934 (Digenea: Cryptogonimidae) infecting the Dory Snapper *Lutjanus fulviflamma* (Teleostei: Lutjanidae) from the east coast of South Africa

**DOI:** 10.1007/s11230-023-10116-1

**Published:** 2023-10-16

**Authors:** Russell Q -Y. Yong, Storm B. Martin, Nico J. Smit

**Affiliations:** 1 Water Research Group, Unit for Environmental Sciences & Management, North-West University, Potchefstroom, 2520 South Africa; 2 Centre for Sustainable Aquatic Ecosystems, Harry Butler Institute, Murdoch University, 90 South Street, Murdoch, WA 6150 Australia

## Abstract

Parasitological assessment of marine fishes at Sodwana Bay in the iSimangaliso Marine Protected Area on the east coast of South Africa revealed a new species of cryptogonimid trematode infecting the pyloric caeca of the Dory Snapper, *Lutjanus fulviflamma* (Forsskål) (Lutjanidae). The new species is morphologically consistent with the concept of the large genus *Siphoderina* Manter, 1934; its phylogenetic position within this genus was validated through molecular sequencing of the ITS2 and partial 28S ribosomal DNA sub-regions. We name this species *Siphoderina nana* **n. sp.** and comment on the current state of understanding for this genus of cryptogonimids.

## Introduction

Research on the fish helminth fauna of South Africa throughout most of the twentieth century has been patchy, with studies being few and far between (Fantham, 1938; Prudhoe, 1956). In the 1970s and 1980s, key work by the likes of Bray (1974, 1978, 1984, 1985, 1987), Gibson (1983) and Gavrilyuk-Tkachuk and Nikolaeva (Gavrilyuk-Tkachuk, 1979; Nikolaeva & Tkachuk, 1979) greatly expanded knowledge on the helminth fauna of South Africa, but research, especially from within South Africa, continued to be relatively rare. As of 2022, just over 70 species of trematode were known from South African waters; this number compares unfavourably with regions of similar latitudes, such as the south coast of Australia (190 recorded species) and subtropical eastern China (529 recorded species) (Bray et al., 2016; Cribb et al., 2016). Most recent aquatic helminthological work in the country has primarily focused on trematodes in freshwater systems (Dos Santos et al., 2021; Hoogendoorn et al., 2020; Malatji & Mukaratirwa, 2019; Rakgole et al., 2019), and then mostly on intermediate stages, with a minority of studies concerning trematodes in marine systems (Huston et al., 2021; Vermaak et al., 2021). Just two new species of fish-infecting trematodes have been described from South Africa in the twenty-first century, one from freshwater (Dos Santos et al., 2021) and one from the marine environment (Vermaak et al., 2023).

Parasitological assessment of fishes from Sodwana Bay on the east Coast of South Africa yielded samples of several trematode species, including a species of cryptogonimid from the Dory Snapper, *Lutjanus fulviflamma* (Forsskål) (Lutjanidae). The Cryptogonimidae Ward, 1917, is a large trematode family with 79 recognised genera and over 280 recognised species (WoRMS, 2023), which, as sexual adults, infect a wide range of (primarily demersal, predatory) freshwater and marine fishes, crocodilians and some snakes and amphibians (Martin et al., 2023; Miller & Cribb, 2008b). Despite their widespread distribution and expansive host range, this is the first cryptogonimid species to be reported from South Africa.

## Methods

Dory Snapper (*L. fulviflamma*) were collected by rod and reel from off Jesser Point, Sodwana Bay in the iSimangaliso Marine Protected Area National Park, South Africa. Following capture, fish were transported in aerated water from the collection site to the nearby field station where they were humanely killed by percussive stunning followed by cranial pithing, and dissected per the protocols of Cribb & Bray (2010). Any worms found were killed and heat-fixed in near-boiling saline, and preserved in 70% ethanol. Specimens for morphological analysis were prepared using standard protocols for Mayer’s haematoxylin preparations, per Yong et al. (2016). All measurements were made using an Olympus SC50 digital microscope camera, mounted on an Olympus BX-53 light microscope and cellSens™ version 1.13 imaging software. All measurements, unless otherwise stated, are in micrometres (μm) and given as a range with the mean in parentheses. Drawings were made with the aid of a *camera lucida* attachment mounted on the same Olympus BX-53 light microscope and digitised in Adobe Illustrator 6.0. Specimens for scanning electron microscopy were prepared by chemical dehydration, first in a graded ethanol series followed by a graded hexamethyldisilazane (HMDS) series, then sputter-coated with gold and photographed using a Phenom Pro Desktop scanning electron microscope (ThermoScientific, Waltham, Massachusetts, USA). Type-specimens have been deposited in the National Museum Parasite Collection of the National Museum, Bloemfontein, South Africa (NMB).

Genetic sequence data were generated from the novel material for cytochrome *c* oxidase subunit 1 mitochondrial marker (*cox*1 mtDNA), the non-coding second internal transcribed spacer unit of ribosomal DNA (ITS2 rDNA), and the large ribosomal subunit gene (28S rDNA). Genomic DNA was extracted using a DNeasy blood and tissue extraction kit (QIAGEN, Hilden, Germany) as per the manufacturer’s instructions, although extractions were eluted once only with 60 µl (*vs* recommend once to twice with 200 µl). The three target marker regions were amplified by conventional PCR, with 25 µl reaction volumes, comprising 2 µl unquantified extracted DNA, 1 µl per primer at 10 µM, 6 µl cresol-red visualisation dye, 9.3 µl purified injection water, 1 µl dNTPs at 4 mM, 2 µl MgCl_2_ at 25 mM and 0.2 µl *Taq*. DNA polymerase at 5.5 units/µl with 2.5 µl 10× reaction buffer (Fisher Biotec Australia), using the primers and cycle schedules provided in Yong et al. (2016) for rDNA and Wee et al. (2017) for mtDNA. These protocols yield a partial read for *cox*1 mtDNA, a full read for ITS2 rDNA with partial flanking 5.8S and 28S, and a partial read for 28S rDNA including the variable domains D1–D3.

Amplicons were visualised via electrophoresis using 1.5% agarose gels stained with SYBR Safe (Invitrogen, California, USA) and purified using Agencourt AMPure magnetic bead purification system (Beckman Coulter, California, USA). Sanger sequencing was completed by the Western Australian State Agricultural Biotechnology Centre (SABC) at Murdoch University, using an ABI Prism™ BigDye v3.1 Cycle Sequencing Kit (Applied Biosystems, California, USA) and an ABI 3730 96 capillary machine. Forward and reverse DNA strands were sequenced using the amplification primers for ITS2 rDNA and *cox*1 mtDNA, whereas the internal primers 300F and 1200R were used for sequencing the 28S rDNA marker; these primers are also detailed in Yong et al. (2016). Contiguous sequences were assembled, in Geneious™ version 10.2.2 (Kearse et al., 2012). The ITS2 region was determined by annotation through the ITS2 Database using the ‘Metazoa’ model (Ankenbrand et al., 2015).

Novel ITS2 and partial 28S rDNA sequences were aligned and analysed against comparable sequence data for other cryptogonimid taxa available on GenBank (see Table 1). As verified *cox*1 mtDNA sequence data are only publicly available for five cryptogonimid taxa, no alignment or analysis of the *cox*1 region was performed. Analyses of the ITS2 rDNA dataset were carried out using only sequences for species of Cryptogonimidae to verify the generic affiliation of our new taxon. Selected species of Heterophyidae Leiper, 1909 + Opisthorchiidae Looss, 1899 were used as outgroup taxa in alignments of the partial 28S rDNA dataset, as these are the closest families to the Cryptogonimidae, as indicated by large-scale molecular phylogenetic analyses by Olson et al. (2003) and Pérez-Ponce de León & Hernández-Mena (2019). Both alignments were performed using MUSCLE 3.7 (Edgar, 2004) with ClustalW sequence weighting and UPGMA clustering for iterations 1 and 2. The resultant alignments were refined by eye using MESQUITE (Maddison & Maddison, 2017) with the ends trimmed to a length matching over 75% of sequences per Yong et al. (2021) and indels greater than three consecutive characters and affecting more than 5% of sequences removed.

**Table 1:** Sequence data from GenBank included in this study

Species	Host species	GenBank accession no. (28S rDNA)	Reference
**Family Cryptogonimidae**			
*Acanthostomum burminis* (Bhalerao, 1926)	*Fowlea piscator* (Colubridae)*	KC489791	Jayawardena et al. (2013)
*Adlardia novaecaledoniae* Miller, Bray, Goiran, Justine & Cribb, 2009	*Nemipterus furcosus* (Nemipteridae)	FJ788496	Bray et al. (2009)
*Anisocladium fallax* (Looss, 1902)	*Uranoscopus scaber* (Uranoscopidae)	KY978883	Kvach et al. (2017)
*Anisocoelium capitellatum* (Rudolphi, 1819)	*Uranoscopus scaber*	KY978882	Kvach et al. (2017)
*Aphalloides coelomicola* Dollfus, Chabaud & Golvan, 1957	*Knipowitschia caucasica* (Gobiidae)	KJ162159	Stoyanov et al. (2015)
*Beluesca littlewoodi* Miller & Cribb, 2007	*Plectorhinchus gibbosus* (Haemulidae)	EF566867	Miller & Cribb (2007c)
*Beluesca longicolla* Miller & Cribb, 2007	*Plectorhinchus gibbosus*	EF566868	Miller & Cribb (2007c)
*Caecincola parvulus* Marshall & Gilbert, 1905	*Micropterus salmoides* (Centrarchidae)	AY222231	Olson et al. (2003)
*Caulanus thomasi* Miller & Cribb, 2007	*Lutjanus bohar* (Lutjanidae)	EF428144	Miller & Cribb (2007b)
*Centrovarium lobotes* (MacCallum, 1895)	*Perca flavescens* (Percidae)	EF547547	Unpublished
*Chelediadema marjoriae* Miller & Cribb, 2007	*Diagramma labiosum* (Haemulidae)	EF566866	Miller & Cribb (2007c)
*Euryakaina marina* (Hafeezullah & Siddiqi, 1970)	*Lutjanus fulviflamma* (Lutjanidae)	HM056037	Miller et al. (2010b)
*Gynichthys diakidnus* Miller & Cribb, 2009	*Plectorhinchus gibbosus*	FJ907333	Miller & Cribb (2009)
*Latuterus maldivensis* Miller & Cribb, 2007	*Lutjanus bohar*	EF428146	Miller & Cribb (2007b)
*Latuterus tkachi* Miller & Cribb, 2007	*Lutjanus bohar*	EF428145	Miller & Cribb (2007b)
*Lobosorchis polygongylus* Miller, Downie & Cribb, 2009	*Lutjanus gibbus* (Lutjanidae)	FJ154902	Miller et al. (2009)
*Lobosorchis tibaldiae* Miller & Cribb, 2005	*Neoglyphidodon melas* (Pomacentridae)^#^	FJ154901	Miller et al. (2009)
*Metadena lutiani* (Yamaguti, 1942)	*Lutjanus bohar*	KF417630	Miller & Cribb (2013)
*Mitotrema anthostomatum* Manter, 1963	*Cromileptes altivelis* (Serranidae)	AY222229	Olson et al. (2003)
*Neochasmus umbellus* Van Cleave & Muller, 1932	*Morone chrysops* (Moronidae)	KY978881	Kvach et al. (2017)
*Neometadena ovata* (Yamaguti, 1952)	*Lutjanus carponotatus* (Lutjanidae)	EF116616	Miller & Cribb (2007a)
*Oligogonotylus mayae* Razo-Mendivil, Rosas-Vaidez & Perez-Ponce de Leon, 2008	*Cichlasoma urophthalmum* (Cichlidae)	MG383507	Martínez-Aquino et al. (2017)
*Pseudosellacotyla lutzi* (Teixeira de Freitas, 1941)	*Hoplias malabaricus* (Erythrinidae)	MH368357	Pantoja et al. (2018)
*Retrovarium brooksi* Miller & Cribb, 2007	*Lutjanus bohar*	EF116605	Miller & Cribb (2007a)
*Retrovarium exiguiformosum* Miller & Cribb, 2007	*Symphorus nematophorus* (Lutjanidae)	EF116612	Miller & Cribb (2007a)
*Retrovarium formosum* Miller & Cribb, 2007	*Symphorus nematophorus*	EF116611	Miller & Cribb (2007a)
*Retrovarium manteri* Miller & Cribb, 2007	*Lutjanus argentimaculatus* (Lutjanidae)	EF116604	Miller & Cribb (2007a)
*Retrovarium planum* Miller & Cribb, 2007	*Symphorus nematophorus*	EF116614	Miller & Cribb (2007a)
*Siphodera vinaledwardsii* (Linton, 1901)	*Sciaenops ocellatus* (Sciaenidae)	AY222230	Olson et al. (2003)
*Siphoderina grunnitus* Miller & Cribb, 2008	*Plectorhinchus gibbosus*	EU571261	Miller & Cribb (2008a)
*Siphoderina hirastricta* (Manter, 1963)	*Lutjanus argentimaculatus*	EU571260	Miller & Cribb (2008a)
*Siphoderina hustoni* Martin & Cutmore, 2022	*Lutjanus rivulatus* (Lutjanidae)	OM721660	Martin & Cutmore (2022)
*Siphoderina infirma* Miller & Cribb, 2008	*Lutjanus russellii* (Lutjanidae)	EU571264	Miller & Cribb (2008a)
*Siphoderina jactus* Miller & Cribb, 2008	*Lutjanus carponotatus*	EU571263	Miller & Cribb (2008a)
*Siphoderina poulini* Miller & Cribb, 2008	*Lutjanus argentimaculatus*	EU571267	Miller & Cribb (2008a)
*Siphoderina quasispina* Miller & Cribb, 2008	*Lutjanus fulviflamma*	EU571265	Miller & Cribb (2008a)
*Siphoderina subuterus* Miller & Cribb, 2008	*Lutjanus adetii* (Lutjanidae)	EU571266	Miller & Cribb (2008a)
*Siphoderina territans* Miller & Cribb, 2008	*Lutjanus carponotatus*	EF116615	Miller & Cribb (2007a)
*Siphoderina virga* Miller & Cribb, 2008	*Lutjanus russellii*	EU571262	Miller & Cribb (2008a)
*Siphomutabilus gurukun* (Machida, 1986)	*Caesio cuning* (Caesionidae)	KF417631	Miller & Cribb (2013)
*Varialvus charadrus* Miller, Bray, Justine & Cribb, 2010	*Lutjanus vitta* (Lutjanidae)	HM187778	Miller et al. (2010c)
**Family Heterophyidae**			
*Centrocestus formosanus* (Nishigori, 1924)	*Homo sapiens* (Hominidae)^†^	KY351633	Le et al. (2017)
**Family Opisthorchiidae**			
*Clonorchis sinensis* Looss, 1907	*Homo sapiens*^†^	JF823989	Thaenkham et al. (2011)
*Opisthorchis viverrini* (Poirier, 1886)	*Homo sapiens*^†^	JF823990	Thaenkham et al. (2011)

Hosts marked with a symbol are non-fish, reptile (*) or mammal (^†^) hosts, or hosts from which the sequenced specimens were recovered as metacercariae (^#^)

The ITS2 and partial 28S rDNA alignments were subjected to Bayesian inference and maximum likelihood phylogenetic analyses to explore affinities of the novel material using MrBayes v3.2.2 (Ronquist et al., 2012) and RAxML v7.2.8 (Stamatakis, 2014), respectively, via the CIPRES portal (Miller et al., 2010a). The GTR+I+Γ model of nucleotide substitution evolution was assumed in analyses for both datasets, based on the Akaike Information Criterion (AIC) in jModelTest v2.1.10 (Darriba et al., 2012). Each Bayesian inference analysis was run over 10,000,000 generations (ngen = 10,000,000) with two runs each containing four simultaneous Markov Chain Monte Carlo (MCMC) chains (nchains = 4) and every 1,000th tree saved (samplefreq = 1000). Bayesian analyses used the following parameters: nst = 6, rates = invgamma, ngammacat = 4, and the priors parameters of the combined dataset were set to ratepr = variable. Samples of substitution model parameters, and tree and branch lengths were summarised using the parameters ‘sump burnin = 3000’ and ‘sumt burnin = 3000’. Maximum likelihood analyses ran 100 bootstrap pseudoreplicates for both datasets.

## Results

### Molecular phylogenetic analyses

**Fig. 1:**
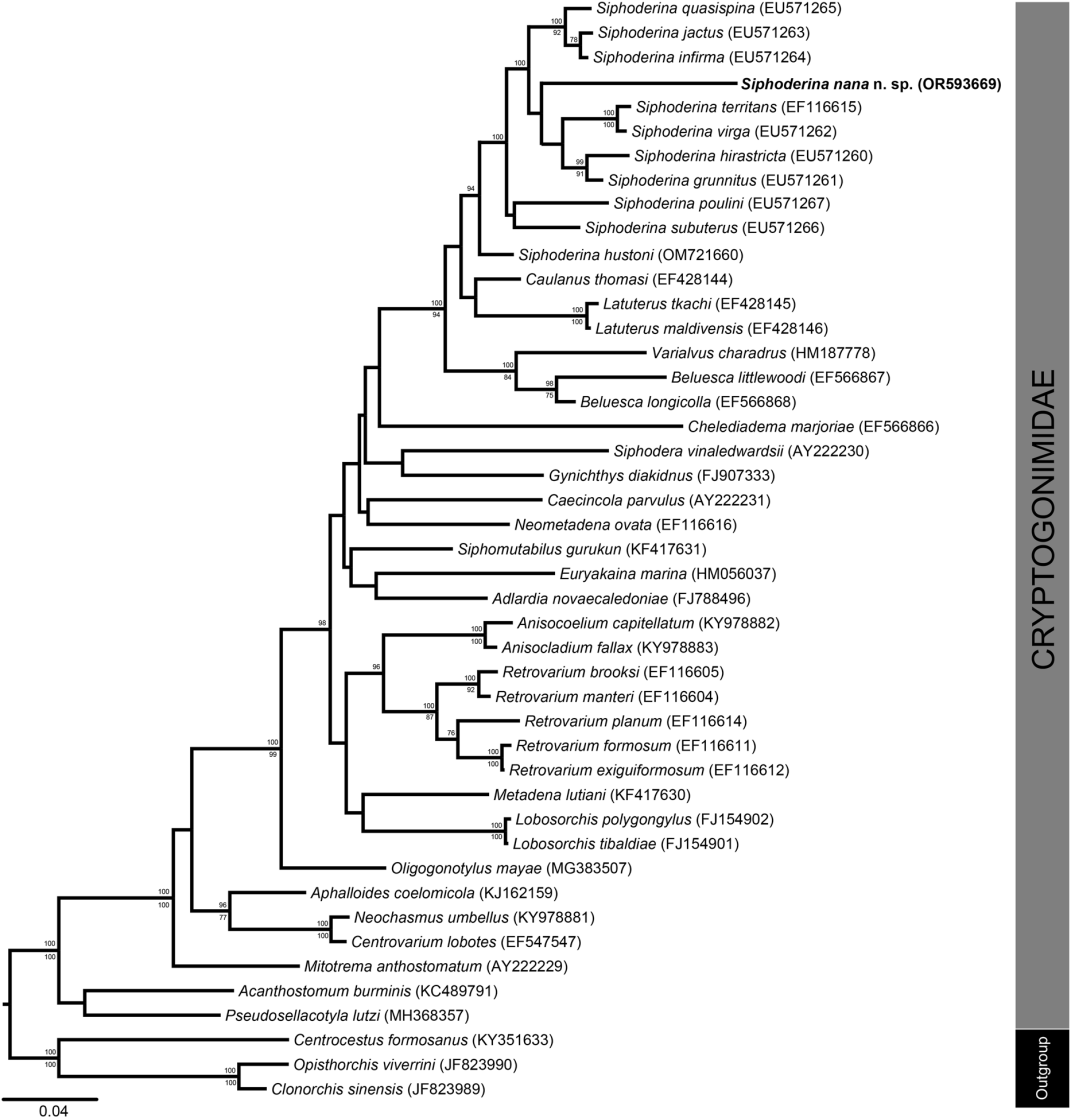
Molecular phylogeny of the Cryptogonimidae, including *Siphoderina nana* **n. sp.** and representatives of other genera for which molecular data were available, based on Bayesian inference and maximum likelihood analyses of the partial 28S rDNA dataset generated by this study (presented topology based on the Bayesian inference analysis), with posterior probabilities from the Bayesian inference analysis shown above the nodes and bootstrap support values from the maximum likelihood analysis below. Nodal support of less than 75 has been excluded.

Alignment of the novel ITS2 rDNA sequence data with those of other cryptogonimid taxa available on GenBank yielded 491 characters for analysis. Bayesian inference and maximum likelihood analyses of the ITS2 rDNA alignment produced trees of essentially identical topologies, with lower nodal support in the latter; these trees are not presented. Alignment of the novel partial 28S rDNA sequence data with those of other cryptogonimid taxa available on GenBank yielded 861 characters for comparative analysis. Bayesian inference and maximum likelihood analyses of the partial 28S alignment produced trees with near-identical topologies (Fig. 1).

In both ITS2 and partial 28S analyses, the novel sequences formed a clade with those of species of *Siphoderina* Manter, 1934, with high nodal support. Our trees largely resemble those for the family produced by Martin & Cutmore (2022) and Miller et al. (2018), with the clade formed by species of *Siphoderina* sister to that formed by species of *Latuterus* Miller & Cribb, 2007, but with disagreement regarding the position of *Caulanus thomasi* Miller & Cribb, 2007. In the ITS2 tree, our new species formed a clade with *Siphoderina hirastricta* (Manter, 1963), sister to *Siphoderina poulini* Miller & Cribb, 2008, but with low confidence at all nodes. In the partial 28S tree, our new species was basal to a clade formed by *Siphoderina territans* Miller & Cribb, 2008 + *S*. *virga* Miller & Cribb, 2008 + *S*. *hirastricta* + *S*. *grunnitus* Miller & Cribb, 2008.

### Taxonomy

**Cryptogonimidae Ward, 1917**

***Siphoderina*** **Manter, 1934**

**Type-species:** ***Siphoderina brotulae*** **Manter, 1934, by original designation**

**Fig. 2:**
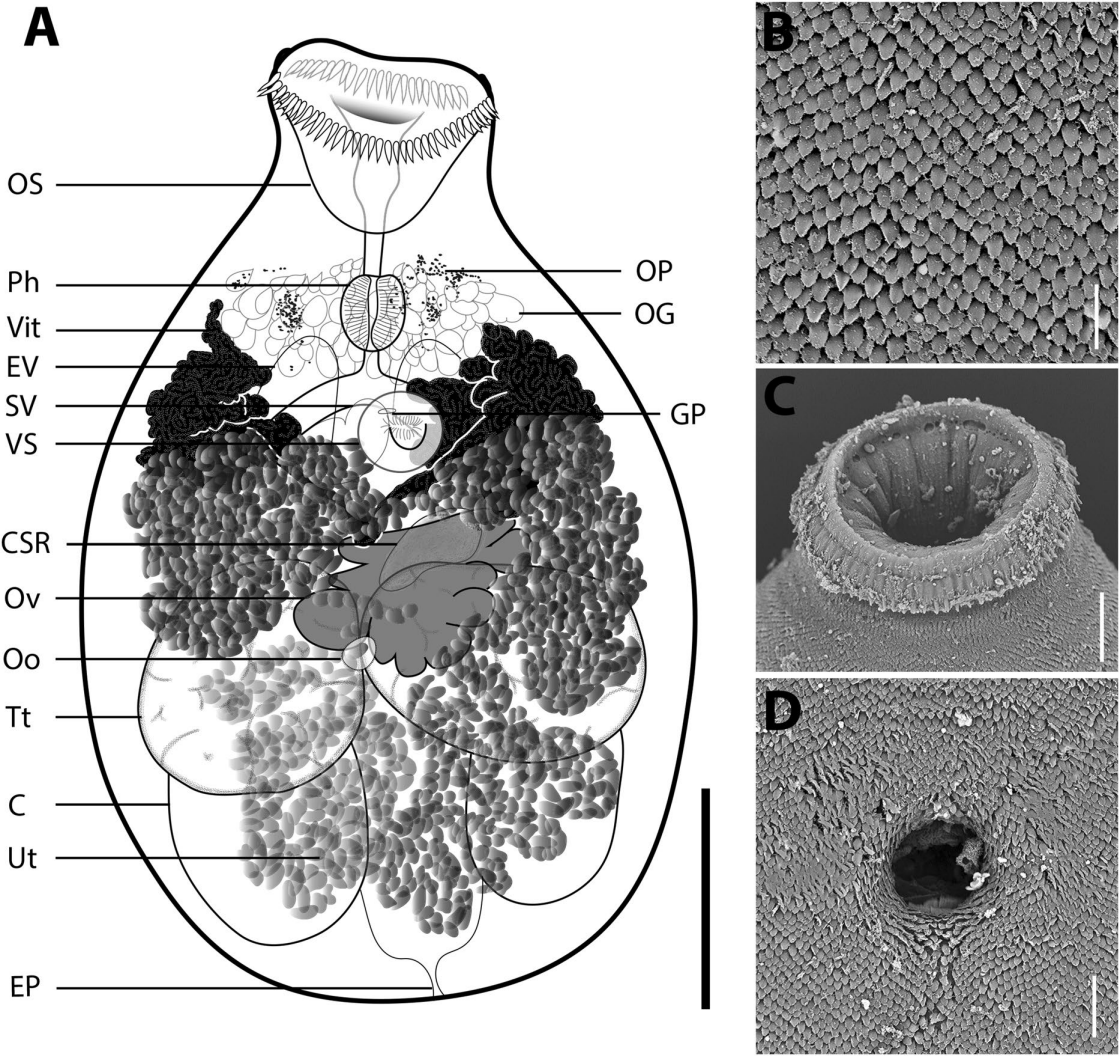
*Siphoderina nana* **n. sp.** **A** Whole-body mount, ventral view of holotype specimen XXX; **B**–**D** scanning electron micrographs of the **B** oral sucker, **C** tegumental spines; and **D** ventrogenital sac opening. *C* caecum, *CSR* canalicular seminal receptacle, *EP* excretory pore, *EV* excretory vesicle, *GP* genital pore (opens internally within ventrogenital sac), *OG* oesophageal glands, *Oo* oötype, *OP* optical pigment, *OS* oral sucker, *Ph* pharynx, *SV* seminal vesicle, *Tt* testis, *Ut* uterus, *Vit* vitelline follicles, *VS* ventral sucker (within ventrogenital sac). Scale-bars: **A** 150 µm, **B** 25 µm, **C** 10 µm, **D** 15 µm.

***Siphoderina nana* n. sp. ** Fig. 2A–D

**Type-host**: *Lutjanus fulviflamma* (Forsskål) (Lutjaniformes: Lutjanidae), Dory Snapper.

**Infection site**: Pyloric caeca.

**Type-locality**: Jesser Point, Sodwana Bay, (27° 32′ 19′′ S, 32° 40′ 48′′ E), iSimangaliso Marine Protected Area, KwaZulu-Natal Province, South Africa.

**Prevalence and intensity**: Three of nine *L*. *fulviflamma* infected with 3–30 worms.

**Material examined**: Holotype (NMB P 970) and 21 paratypes (NMB P 971–990), all slide-mounted.

**Representative DNA sequences**: Three identical replicates of ITS2 rDNA, 466 nucleotides, one submitted to GenBank (OR593670); two identical replicates of partial 28S rDNA, 1,121 nucleotides, one submitted to GenBank (OR593669); three sequences of *cox*1 mtDNA comprising two genotypes varying at 1 of 491 nucleotides (OR590623 representative of two replicates, and OR590624 representative of one replicate).

**ZooBank registration**: The species *Siphoderina nana* is registered in ZooBank under urn:lsid:zoobank.org:act:C29020A4-6535-4F31-B4A1-B623AC1FE964.

**Etymology**: The specific name *nana* is a feminine Latin noun, itself derived from the Ancient Greek “*nânos*”, meaning “dwarf”, as it is the smallest known species of the genus *Siphoderina*.

### Description

[Based on 22 whole-mounted, unflattened specimens]. Body usually sub-globular, occasionally pyriform when oral sucker extended, 385–692 × 239–458 (547 × 357), 1.2–1.8 (1.5) times longer than wide. Tegumental spines flattened, broadly lanceolate, with serrated edges, arranged in the manner of overlapping scales, evenly covering whole of body except oral sucker, 5 long (Fig. 2B). Forebody 140–254 (182) long, represents 27.7–38.3% (33.6%) of body length. Remnant eyespot pigment present in some specimens; dispersed in forebody. Oral sucker infundibuliform, 70–126 × 103–161 (98 × 128), 0.5–1.2 (0.8) times longer than wide. Oral spines 60–72 (67), straight-tipped, ringing periphery of oral sucker, 14–16 (15) long (Fig. 2C). Ventral sucker distinctly smaller than oral sucker, spherical, set medially on ventral body surface within ventrogenital sac [per Miller & Cribb (2008a)], 41–61 × 42–60 (51 × 52), 0.8–1.2 (1.0) times longer than wide. Ventrogenital sac opens medially; aperture 10–22 (15) across, circular; gonotyl or other distinct tegumental folding absent (Fig. 2D). Prepharynx 11–40 (26). Pharynx ellipsoidal, similarly sized to ventral sucker, 39–60 × 36–47 (48 × 41), 1.0–1.5 (1.2) times longer than wide. Oesophagus straight, 11–32 (19). Gland cells, probably associated with mouth or pharynx, profuse, pyriform, centred on oesophagus, distributed throughout forebody to level of caecal bifurcation. Intestinal bifurcation medial, in space between pharynx and ventral sucker. Intestinal caeca blind-ended, straight, extremities often greatly expanded, pass parallel to lateral body margins, 220–503 (366) long, span 52.0–80.0% (66.6%) of total body length, terminate 35–115 (68) from posterior extremity of body; post-caecal zone 14–88 (44) or 3.5–18.4% (8.3%) of total body length.

Testes two, spherical to sub-spherical, opposite to slightly oblique, in mid-hindbody, left testis 93–248 × 93–209 (152 × 151); right testis 77–242 × 108–208 (155 × 151); pre-testicular zone 201–368 (292) or 45.6–61.5% (51.7%) of total body length; post-testicular zone 59–202 (127) or 13.1–29.3% (22.8%) of total body length. Seminal vesicle medial, saccular, usually deflexed, highly contorted in some specimens, 77–194 × 24–75 (118 × 47). Ejaculatory duct passes dorsal to ventral sucker, path almost entirely obscured by uterine eggs. Genital atrium obscured by ventral sucker, varying from almost indiscernible to very short, simple. Genital pore medial, opens antero-dorsal to ventral sucker within ventro-genital sac, 148–246 (192) or 29.5–41.3% (35.4%) of total body length from anterior extremity of body and 214–474 (340) or 55.6–68.8% (61.8%) from posterior extremity.

Ovary an irregularly-shaped, highly lobulate mass, asymmetrically medial in anterior hindbody between ventral sucker and testes, posteriorly overlaps anterior margins of testes, 75–219 × 67–237 (117 × 158). Canalicular seminal receptacle sub-medial, oval to oblate trapezoid, antero-dorsal to ovary, size and volume varying with sperm content, 34–120 × 54–155 (61 × 82). Oötype not discernible in most specimens, medial between testes, immediately posterior to ovary. Mehlis’ gland not discernible. Laurer’s canal not detected. Uterine coils convoluted and densely packed with eggs, commence immediately posterior to oötype, pass posteriad to occupy much of post-ovarian space, then ascend antero-sinistrally to level of ventral sucker and then dextro-laterally to occupy most of space between intestinal bifurcation and testes; distal-most coil ascends antero-medially to meet genital atrium. Metraterm absent. Eggs ovoid, translucent and lightly tanned in proximal uterine coils, gradually darkening as they mature within uterus, darkest in distal/anterior-most uterine coils, 15–16 × 8–9 (16 × 8). Vitellarium comprises a pair of densely clumped lateral fields that extend anteriorly to level of pharynx up to 94–185 (140) from anterior extremity and posteriorly to level of ovary, occasionally medially confluent, dorsal to uterine coils, appear to surround caeca and excretory vesicle. Vitelline ducts extend postero-diagonally from vitelline fields, meet medially in mid-body posterior to ventral sucker. Excretory vesicle Y-shaped, bifurcates in mid-hindbody postero-dorsal to ovary; arms vary greatly in volume, extend to level of pharynx.

### Differential diagnosis

A combination of ovoid-to-fusiform body shape, smooth-margined testes opposite (as opposed to distinctly oblique or in tandem) in the anterior hindbody, and uterine coils that are extensively distributed pre- and post-testicular, and reach but do not exceed the level of the ventral sucker, differentiates *Siphoderina nana* **n. sp.** from all but nine of the 49 recognised species of *Siphoderina*: *S*. *asiatica* Gu & Shen, 1979, *S*. *ghanensis* (Fischthal & Thomas, 1968), *S*. *grunnitus*, *S*. *marangsi* Machida, 2009, *S*. *provitellosa* (Durio & Manter, 1969), *S*. *ramadani* (Nahhas, Sey & Nishimoto, 1998), *S*. *sootai* (Hafeezullah, 1975), *S*. *territans*, and *S*. *ulaula* (Yamaguti, 1970). Of these nine, eight infect species of lutjanid fishes, the exception being *S*. *grunnitus*, which is known only from the haemulid *Plectorhinchus gibbosus* (Lacépède). *Siphoderina nana* is smaller than all other *Siphoderina* species, with a maximum length of only 692 µm, distinctly smaller than the minimum size of *S. asiatica* (952 µm), *S. ghanensis* (899 µm), *S. marangsi* (800 µm), *S. provitellosa* (992 µm), *S. sootai* (1,112 µm) and *S. ulaula* (2,200 µm). *Siphoderina nana* also has more oral sucker spines (60–72) than *S. marangsi* (20–28) and *S. ulaula* (42–58), and fewer than *S. provitellosa* (~135).

In having 60–72 oral spines, *Siphoderina nana* is similar to *S. asiatica* (65–74), *S. ghanensis* (70–75), *S. ramadani* (up to 60) and *S. territans* (53–66); the number of oral spines in *S. sootai* is unknown. Of those, *S. nana* can be immediately differentiated from *S. asiatica*, *S. ghanensis* and *S. sootai* by body size. Unlike *S. nana*, all three of those species show no overlap between the ovary and the testes; the ovary of *S. asiatica*, in particular, appears stellate in shape rather than irregularly lobed. The former two species also show more elongate, fusiform body shapes, with a higher ratio of length to breadth: ~ 1.9 and 2.1 times longer than broad, respectively, versus 1.2–1.8 (mean 1.5) times for *S. nana. S. territans* overlaps with *S. nana* in body size and oral spine count, as well as having an irregularly lobed ovary which extensively overlaps the testes, extensive pre-testicular uterine coils and similar vitelline follicle distribution. It is, however, larger than *S. nana*, averaging 706 µm long (range 533–946 µm), has a greater body length/breadth ratio (1.6–2.4, mean 2.0) and larger eggs (18–23 × 8–11, mean 20 × 9, *vs* 15–16 × 8–9, mean 16 × 8). The host range of *S. territans* is so far limited to *Lutjanus carponotatus* (Richardson), whereas *S. nana* infects *L. fulviflamma*. The two species are also genetically distinct from one another, differing by 28 bp and 32 bp in the ITS2 and 28S rDNA regions respectively. *Siphoderina nana* most closely resembles *S. ramadani* and the two are known from the same host. However, *S. ramadani* is larger than *S. nana*, with a minimum body size of 691 µm (max 1408, mean 906) whereas the maximum size of *S. nana* is 692 µm, and the number of oral spines described for *S. ramadani* was “up to 60” (Nahhas et al., 1998) whereas the minimum number of oral spines for *S. nana* is 60. Obtaining molecular sequence data for *S. ramadani* to clarify its phylogenetic position, particularly in relation to *S. nana* **n. sp.**, is desirable.

## Discussion

Our description of *Siphoderina nana* **n. sp.** brings the number of species in this genus to 49. The taxonomic history of this genus has been complex since its original proposition by Manter (1934) for *S*. *brotulae* from the ophidiid *Brotula barbata* (Bloch & Schneider). Species of several genera (*Metadena* Linton, 1910, *Neochasmus* Van Cleave & Müller, 1932, *Paracryptogonimus* Yamaguti, 1934, *Lappogonimus* Oshmarin, Mamaev & Parukhin, 1961 and *Pseudallacanthochasmus* Velasquez, 1961) have been subsequently transferred to *Siphoderina* (Miller & Cribb, 2008a, b). The majority of species (35 of 49) infect lutjanids, five infect the ecologically-similar centropomids and a further two infect haemulids, which are phylogenetically most closely related to lutjanids (Betancur-R et al., 2017); two species, *S*. *ghanensis* and *S. grandispinus* (Velasquez, 1961), have been recorded from both lutjanids and haemulids (Fischthal & Thomas, 1968; Velasquez, 1961). This leaves a minority of species infecting ecologically and phylogenetically disparate hosts, e.g. species of Gobiidae [*S*. *microvata* (Tubangui, 1928)], Cirrhitidae [*S*. *mexicana* (Bravo-Hollis, 1953)] and Uranoscopidae (*S*. *xenocephali* Machida, 2009) (Bravo-Hollis, 1953; Machida, 2009; Tubangui, 1928).

Several species of *Siphoderina* have been recorded from multiple phylogenetically, geographically and ecologically disparate hosts, further complicating interpretations of true host range within the genus. The type-species, *S*. *brotulae*, for example, was described from a brotula (Ophidiidae), but was subsequently reported infecting snappers (Lutjanidae) and goosefishes (Lophiidae), a host range across three fish orders (Ophidiiformes, Lutjaniformes and Lophiiformes, respectively) (Dyer et al., 1992; Manter, 1934, 1947). *Siphoderina americana* (Manter, 1940) has been recorded from both eastern and western American coasts, in seven lutjanid species (Fischthal, 1977; Manter, 1940), a centropomid (Lamothe-Argumedo, 1969), a batrachoidid (Pearse, 1949) and a serranid (Fischthal, 1977), as well as lutjanids from off Karwar, Karnataka on the west coast of India (Hafeezullah & Siddiqi, 1970). Most remarkably, *S*. *grandispinus*, described by Velasquez (1961) from an unknown *Lutjanus* species from the Philippines and also known from the haemulid *Pomadasys hasta* (Bloch) from off Kochi (Cochin), Kerala in western India (Hafeezullah & Siddiqi, 1970), has also been reported from the freshwater characiform *Hoplias malabaricus* (Bloch) (Erythrinidae) from Porto Alegre, Brazil (Fortes et al., 1996).

In many cases, the accounts in these reports are no more than cursory entries in bigger lists of host-parasite combinations, the result of more general parasitological surveys, with no accompanying descriptions, illustrations or specimen accession information. The overly simple nature of some early descriptions and their accompanying illustrations, and frequent use of excessive flattening during specimen preparation that warps anatomical features, also hinders effective interpretation. Most of these reports pre-date molecular sequencing techniques. Studies which make use of molecular sequence data, like those conducted by Miller & Cribb (2008a), indicate a tendency among species of Cryptogonimidae, including *Siphoderina*, toward high host-specificity. This, in turn, is reflective of a wider trend among trematodes of tropical marine fishes, in which genuinely euryxenic species are comparatively rare, and species once regarded as having broad host ranges on the basis of morphology alone are revealed to be complexes of multiple species when scrutinised using molecular sequencing (Miller et al., 2011). We are therefore sceptical of many of these reports. The few reports with accompanying illustrations leave little doubt that the taxa observed were indeed cryptogonimids, and these taxa may even be closely related to the species they are purported to be. In the absence of available specimens and especially molecular sequence data, we cannot definitively rule on their actual identity. Nevertheless, it seems likely that at least some of these reports of species of *Siphoderina* will prove erroneous.

*Siphoderina* is the largest cryptogonimid genus and among the least distinctive concepts in the family. As such, it currently accommodates substantial morphological variability. For example, considered in isolation, it seems unlikely that the diminutive *S*. *nana*, reaching at most 692 µm long, should be considered a good congener of *S. onaga* (Yamaguti, 1970), which reportedly reaches 11,200–12,500 µm long (Machida, 2009). Nevertheless, in our new analyses, all represented species of *Siphoderina* continue to form a monophyletic clade. Re-collection and sequencing of other *Siphoderina* species, especially the type-species *S. brotulae* and those other than from lutjanids and haemulids, will be informative for determining and refining the composition and definition of the genus. Indeed, although it is possible for species of Cryptogonimidae from a single genus to greatly vary morphologically [see Miller & Cribb (2007a, 2013)], for at least a few of the most morphologically disparate taxa, we suspect they will prove to not be close relatives of “good” species of *Siphoderina* at all. Rather, we suspect that the genus *Siphoderina* might incorporate multiple disparate groups, currently united via an overly generalised concept, and that the highly morphologically divergent forms might represent distinct radiations, possibly corresponding to ecologically or phylogenetically related host groups. Such a pattern is not without precedent among trematodes, e.g. the blood fluke genus *Cardicola* Short, 1953 (Aporocotylidae) (see Yong et al., 2021).

The novel sequence data for *S*. *nana* are the first for a cryptogonimid from South Africa. Prior to the new analyses, sequence data existed for just 10 of the (now) 49 species of *Siphoderina*, all of which were generated from specimens collected in Australian waters (Martin & Cutmore, 2022; Miller & Cribb, 2008a), and indeed the majority of sequences available for cryptogonimids are from Australian taxa. In the new analyses, novel data for *S*. *nana* resolved deep within the *Siphoderina* clade, clearly demonstrating that the Australian taxa do not form a monophyletic clade, i.e. *S*. *nana* is more closely related to some Australian species than those species are to some other Australian congeners. Further collection and sequencing efforts from South Africa and elsewhere in the western Indian Ocean are required to begin to understand the extent to which cryptogonimid species might be shared across the Indo-West Pacific, although, given the considerable efforts in Australia, it would appear that *S*. *nana* at least should be considered unlikely to occur there.

The largest radiation of species of *Siphoderina* has occurred in lutjanid fishes, and several lutjanid species are reported hosts for multiple *Siphoderina* species. The host for *Siphoderina nana*, *L. fulviflamma*, is the reported host for six other species of *Siphoderina*. Gu & Shen (1979) described *S*. *asiatica* from this host, as well as *L*. *sanguineus* (Cuvier), from off Hainan Island, China. Saoud et al. (1988) recorded *S*. *leilae* (Nagaty, 1957) (as *Metadena leilae*) in *L*. *fulviflamma* from the Persian/Arabian Gulf off Qatar. Nahhas et al. (1998) described *S*. *ramadani* in *L*. *fulviflamma* from the Kuwaiti coast, also in the Persian/Arabian Gulf. Nahhas et al. (2003) reported *Siphoderina acanthostomus* (Yamaguti, 1934) (as *Paracryptogonimus acanthostomus* Yamaguti, 1934) from this fish host from Fiji. Most recently, Miller & Cribb (2008a) described two species in *L*. *fulviflamma*, *S*. *jactus* Miller & Cribb, 2008 and *S*. *quasispina* Miller & Cribb, 2008 from Heron Island in the southern Great Barrier Reef, eastern Australia, with *S. quasispina* also found in the same host off northwestern Australia. Thus, *L*. *fulviflamma* is host to the greatest number of *Siphoderina* species of any lutjanid, and indeed any fish. Our phylogenetic analyses do not indicate that all these species represent a single radiation; rather, we infer that species of *Siphoderina* have invaded *L*. *fulviflamma* at least twice. It is unclear why *L. fulviflamma* seemingly supports greater richness of *Siphoderina*, except that this fish is common across its broad range and so has likely been sampled more frequently than most other lutjanids. Further sampling of lutjanids across the Indo-West Pacific, and perhaps especially from the western coast of Africa, will almost certainly continue to uncover substantial further richness of *Siphoderina* species.
